# What Time Is It? It Is 8 and 1/2 Time: A Rare Case of Ischemic Stroke Consistent With Eight-and-a-Half Syndrome

**DOI:** 10.1177/23247096251363013

**Published:** 2025-08-16

**Authors:** Jessica Daza, Marcelo Bedoya-Sommerkamp, Eunbee Cho, Celia Daza, Francisco Gomez, Fatimah Bello, Jia Lin

**Affiliations:** 1 Department of Internal Medicine, The University of Texas Rio Grande Valley (UTRGV), Edinburg, USA; 2 Department of Neurology, The University of Texas Health Science Center at Houston (UTHealth Houston), Houston, USA; 3 Department of Internal Medicine, Universidad Libre de Colombia, Bogotá, Colombia

**Keywords:** eight-and-a-half syndrome, neuro-ophthalmology, ischemic stroke, pontine ischemia, internuclear ophthalmoplegia

## Abstract

Eight-and-a-half syndrome is a rare neuro-ophthalmic syndrome characterized by conjugate horizontal gaze palsy, ipsilateral internuclear ophthalmoplegia, and ipsilateral facial nerve palsy. It results from a lesion affecting the paramedian pontine reticular formation, the medial longitudinal fasciculus, and the abducens nucleus of the same side, at the level of the lower pons. We present a case of a 49-year-old man with 2-week diplopia and 3-day right-sided facial droop, drooling, and dysarthria. CT angiography of the head and neck revealed atherosclerotic disease, with decreased caliber of the basilar artery at the level of the pons. Brain MRI showed a subacute ischemic lesion in the right dorsal pons. The prognosis is favorable with improvement of facial palsy and ocular symptoms. In addition to treating the underlying etiology, neurorehabilitation is indicated. This case highlights the importance of identifying the level of the lesion in patients with gaze palsy and facial droop, as well as correlating the findings into syndromic diagnoses.

## Introduction

The eight-and-a-half syndrome (EHS) is a combination of one-and-a-half syndrome (horizontal-gaze palsy plus internuclear ophthalmoplegia [INO]) and ipsilateral facial nerve palsy. This uncommon syndrome was first described by Eggenberger.^1,2^ It results from a lesion in the dorsal tegmentum of the caudal pons, involving the paramedian pontine reticular formation (PPRF), the medial longitudinal fasciculus (MLF), as well as the nucleus and fasciculus of the facial nerve.^3^

The most common causes of EHS are vascular etiologies such as pontine infarction or ischemia, demyelinating conditions like multiple sclerosis, or, in rarer instances, space-occupying lesions such as tuberculoma. To date, no associations have been found between EHS and race, age, or gender.^4^ Common comorbidities observed among patients with this condition include hypertension, diabetes mellitus, and hypercholesterolemia.

Prognosis depends on the underlying disease entity. In cases involving infarction, neurological outcome is contingent upon the ability of the affected areas to recover. Diplopia and facial palsy show excellent return of function; however, abduction weakness may persist longer than adduction weakness. Neuro-rehabilitation plays a critical role in maximal recovery for the individual diagnosed with EHS.^3^

## Case Presentation

This is a 49-year-old Hispanic man with a medical history of essential hypertension, uncontrolled type 2 diabetes mellitus, hyperlipidemia, and a family history of strokes in first-degree relatives, who came to the emergency department presenting with 2 weeks of double vision associated with 3 days of right-sided facial droop, slurring speech, and drooling.

**Figure 1.**
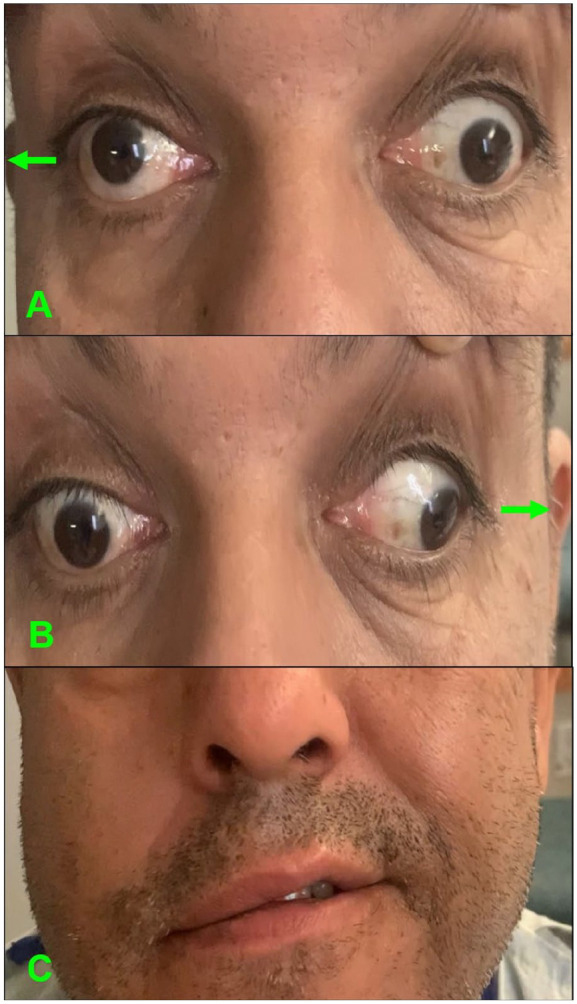
Neurological examination. (A) Patient looking to the right: lateral palsy of the right eye, medial palsy of the left eye. (B) Patient looking to the left: medial palsy of the right eye, abduction of the left eye with nystagmus. (C) Patient smiling: right facial droop extending to the forehead.

Vital signs were remarkable for mildly elevated blood pressure (148/90 mmHg). Neurological examination revealed binocular horizontal diplopia and disconjugated primary gaze with lateral and medial gaze palsy of the right eye and medial gaze palsy of the left eye (Figure 1). A prominent right INO was present. Additionally, right facial droop was evident with incomplete facial activation extending to the forehead, suggesting lower motor neuron involvement (CN VII). Bilateral exotropia and mild dysarthria were also seen. The rest of the exam, including motor and sensory functions, was unremarkable.

**Figure 2.**
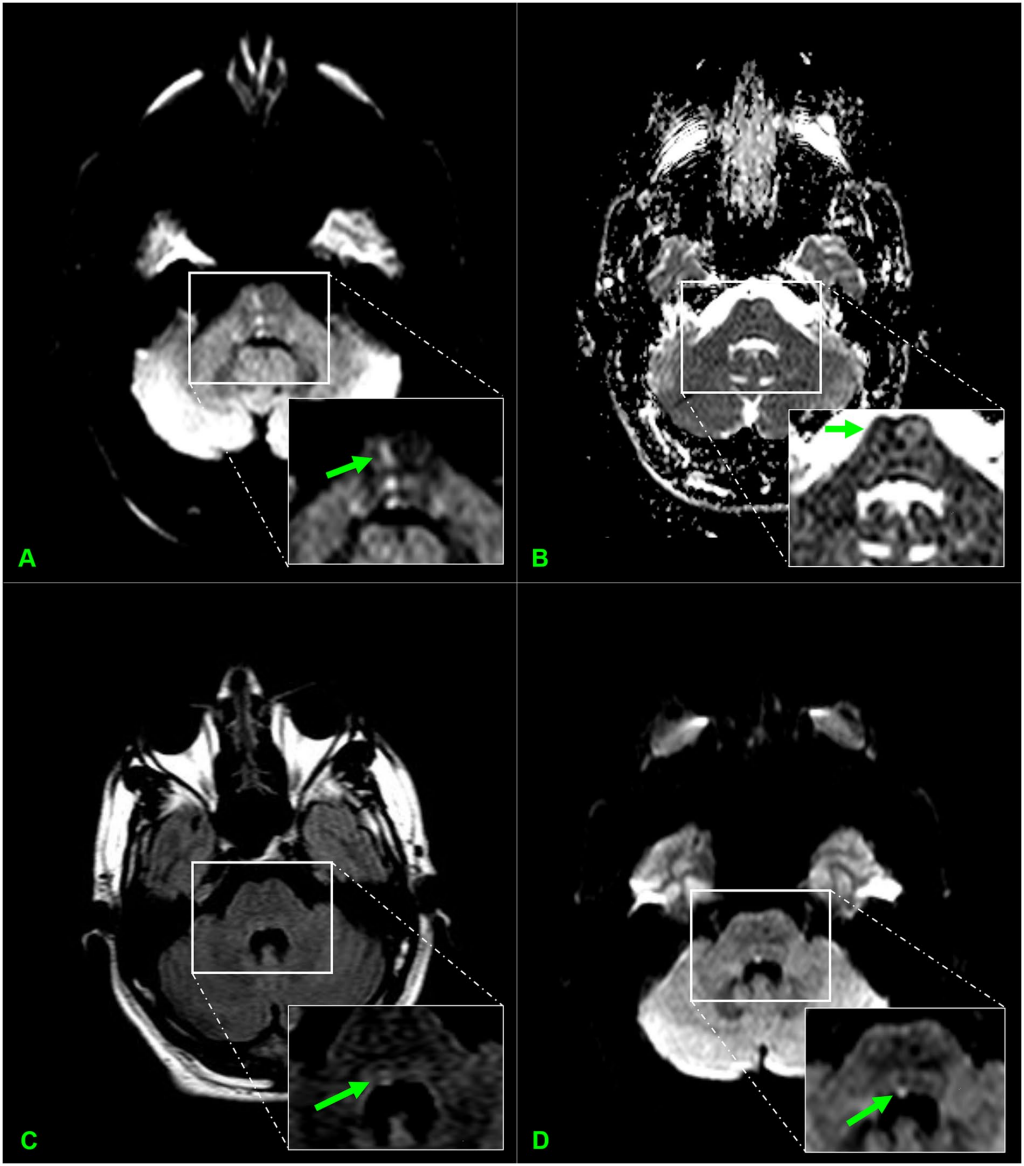
Different MRI sequences showing a subacute ischemic infarct (green arrows). (A) Diffusion-weighted imaging (DWI) showing hyperintensity in the right pons. (B) Apparent Diffusion Coefficient (ADC) showing hypointensity in the right pons. (C) T2 Fluid-Attenuated Inversion Recovery (FLAIR) showing hyperintensity in the right facial colliculus. (D) DWI showing hyperintensity in the right facial colliculus.

Serum evaluation showed elevated HbA1c of 9.4%; ECG, trans-thoracic echocardiogram, and CT head without contrast were unremarkable. CT angiogram of the head and neck showed atherosclerotic disease, with second-degree carotid stenosis (30%-49%) and decreased caliber of the basilar artery at the level of the pons. The abrupt onset of symptoms in the patient suggested an ischemic etiology. In the setting of a family history of strokes at an early age (mother, sister, and brother had strokes before the age of 45), an autoimmune and hypercoagulable workup was performed and was negative (Table 1). A brain MRI showed a subacute ischemic lesion at the level of the right pons and facial colliculus with involvement of the MLF (Figure 2). The patient’s clinical findings, together with the MRI results, were consistent with the diagnosis of EHS.

**Table 1.** Hypercoagulable and Autoimmune Tests Performed in Serum. All Results Were Negative/Within Normal Range.

Test	Result	Test	Result
PT/INR	10.1/0.93	Protein S activity (%)	104
PTT	26.2	Protein S Ab (%)	86
Factor V Leiden mutation	Negative	P-ANCA/C-ANCA	<1:20
Antithrombin III activity (%)	115	Antiphospholipid Ab	Negative
Protein C activity (%)	120	Anticardiolipin Ab IgM/IgG	<10/<10
Protein C Ab (%)	93	β-2 Glycoprotein I Ab IgA/IgM/IgG	<10/<10/<10

Abbreviations: PT, prothrombin time; INR, international normalized ratio; PTT, partial thromboplastin time; Ab, antibody; P-ANCA, perinuclear antineutrophil cytoplasmic antibody; C-ANCA, cytoplasmic antineutrophil cytoplasmic antibody.

The patient was started on aspirin 325 mg, clopidogrel 75 mg, and atorvastatin 80 mg. His glucose and blood pressure were optimized with insulin glargine and lisinopril, respectively. During the hospital course, the patient had no new neurological deficits and was discharged home on daily sitagliptin-metformin XR 50-1000 mg, glipizide 2.5 mg, canagliflozin 100 mg, lisinopril 20 mg, aspirin 81 mg, clopidogrel 75 mg, and atorvastatin 80 mg.

**Figure 3.**
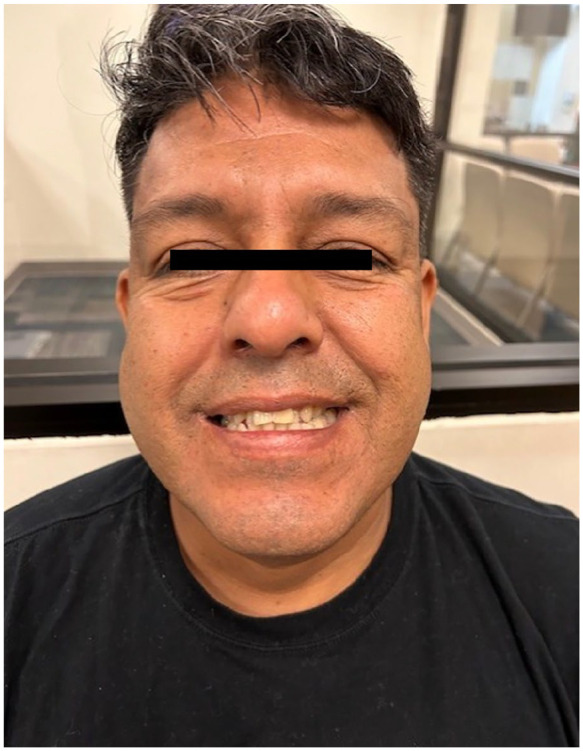
Patient, when smiling 6 months after the initial visit: mild right facial droop extending to the forehead, improved when compared to initial presentation.

The patient followed up with his primary care physician and outpatient neurology. Due to cultural preferences, he had declined physical therapy and opted for a nonconventional herbal massage therapy, consisting of herbal oils, creams, and compresses. However, the patient was compliant with the medical regimen; blood pressure and glucose levels were well controlled. He had mild residual episodic diplopia, which self-corrected upon convergence and mild right facial droop extending to the forehead (Figure 3), but had not presented any new neurological events. (Online supplement – Video 1)

## Discussion

The PPRF is the primary center for horizontal conjugate gaze control and saccades. It is located in the dorsal pons adjacent to the abducens nucleus (CN VI) at the level of the facial colliculus. This structure sends a signal to the ipsilateral CN VI and the contralateral oculomotor nucleus (CN III) through the MLF.^4^ A lesion in the PPRF would cause ipsilateral horizontal conjugate gaze palsy and impaired ipsilateral horizontal saccades, resulting in asynchronous movements of the eyes in one direction.

On the other hand, the MLF receives the signals from the contralateral PPRF and CN VI and transmits to the ipsilateral CN III for coordinated eye movements. It extends across the dorsal tegmentum of the midbrain, pons, and medulla.^5^ A lesion in the MLF would cause abnormal gaze toward the opposite side: defective adduction of the ipsilateral eye and horizontal jerky nystagmus of the contralateral eye on abduction. However, gaze toward the side of the lesion is normal.^4,6^ This is called INO, a disorder of conjugate lateral gaze.^5^

**Figure 4.**
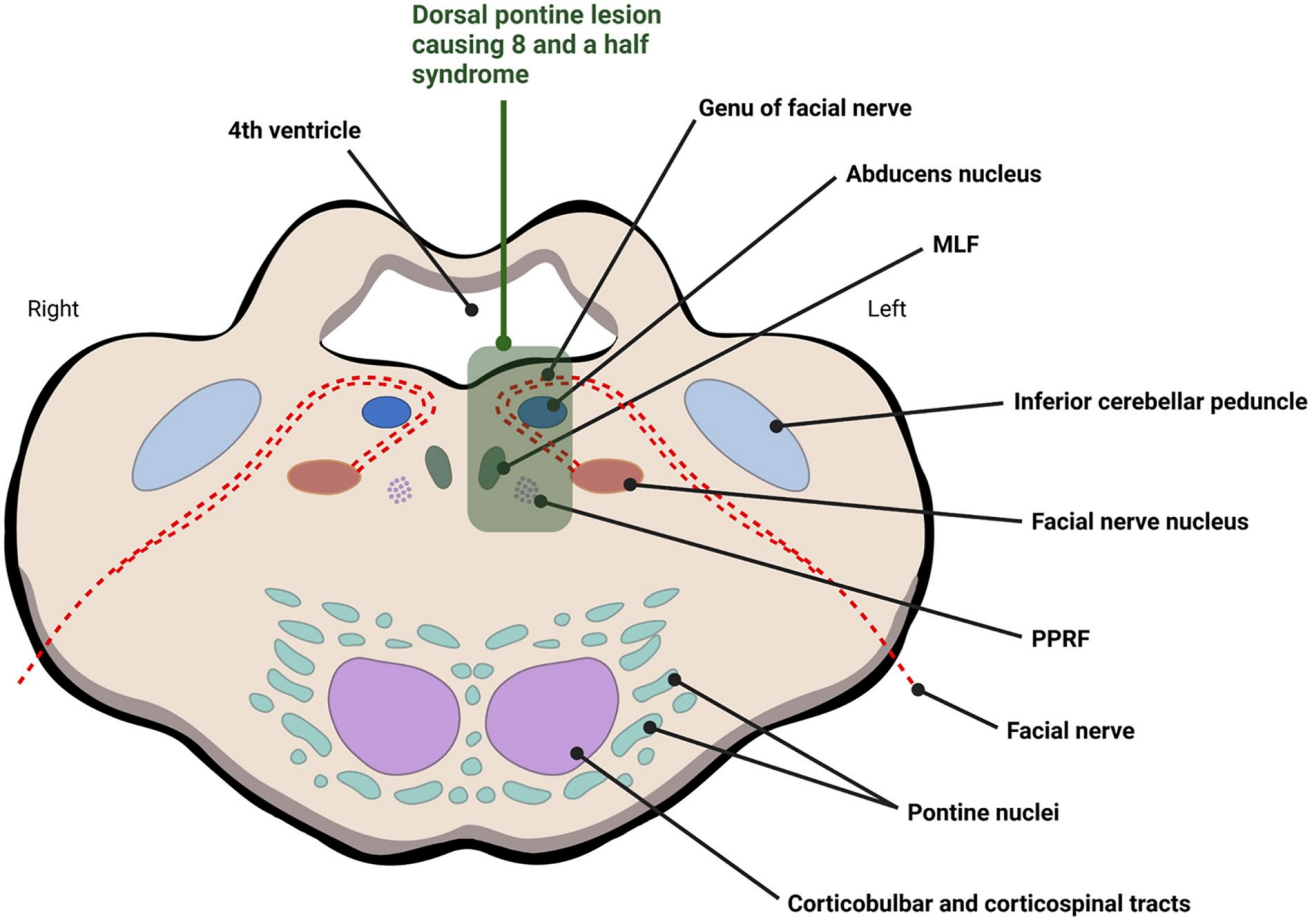
Schematic cross section of the pons showing a lesion at the dorsal medial level causing eight-and-a-half syndrome. Created in BioRender. Bedoya-Sommerkamp, M. (2025) https://BioRender.com/yjwgxgm

When both the PPRF/CN VI and MLF from the same side are damaged, it is called one-and-a-half syndrome (Figure 4). First described by Fisher, this condition consists of ipsilateral conjugate horizontal gaze palsy (“the one”) and ipsilateral INO (the “half”).^4,7^ It is characterized by absent horizontal movements of the ipsilateral eye. The contralateral eye’s abduction is preserved with observed nystagmus, while adduction is absent.^8^ Vertical gaze and convergence are preserved.^3,9^

The EHS is the isolated combination of one-and-a-half syndrome and ipsilateral lower motor neuron facial nerve (CN VII) palsy (1.5 + 7). Eggenberger first described this infrequent neuro-ophthalmic condition in patients with ischemic stroke.^1,2^ So far, 31 cases have been reported in the literature of EHS secondary to an ischemic lesion.^10^

Association with sex, race, or age has not been observed. However, hypertension and diabetes mellitus are the most commonly found in comorbidities patients with this disease.^11^ The damage, regardless of its etiology (vascular, demyelinating, or space-occupying), is anatomically and clinically localized in the dorsal tegmentum of the caudal pons. This unique syndrome can be explained by the proximity of the PPRF, CN VII, and MLF.^1-4,6,12-14^ In our case, the abrupt onset of symptoms in the patient suggested an ischemic stroke, confirmed with neuroimaging.

Blood supply to this area is derived from paramedian pontine arteries, which are small perforating vessels originating from the basilar artery.^12^ Our patient presented with hypertension, uncontrolled type 2 diabetes mellitus, and a family history of first-degree strokes, thereby increasing the risk for a vascular lesion. This was caused by atherosclerosis and subsequently decreased caliber of the basilar artery, as seen on the CT angiogram of the head and neck. Given the origin of the blood supply, hypertension was probably the most important risk factor in this patient.

The EHS is mainly a clinical diagnosis; however, brain MRI can provide insights regarding the underlying etiology.^12^ Our patient’s brain MRI showed multiple small lesions in the right pons and one in the right facial colliculus without enhancement or vasogenic edema (Figure 2), consistent with a subacute ischemic stroke.

Treatment of EHS depends on the underlying etiology in addition to neurorehabilitation. In patients with severe diplopia, an eye patch can be used, alongside lubricating eye drops. The facial palsy is usually addressed with targeted physiotherapy.^11^ Our patient received optimal medical treatment for a subacute ischemic stroke of the posterior circulation, including dual antiplatelet therapy and a high-dose statin. Physical therapy was also indicated to ensure prompt recovery, but the patient declined due to cultural preferences in alternative herbal massage therapy.

The prognosis is usually excellent based on the specific etiology.^3^ In the case of a vascular lesion, the recovery depends on the size of the infarct and the ability of the affected areas to recover.^2^ It is worth noting that mortality rate in pontine stroke ranges from 30% to 50%.^11,15^ In most of the patients, diplopia and facial palsy resolve, whereas weakness in abduction persists longer than adduction. This may be due to the involvement of CN VI fascicle and MLF, rather than the nucleus itself, or may reflect a relative vulnerability of CN VI motor neurons innervating the lateral rectus.^1,3^

## Conclusion

Isolated conjugate horizontal gaze palsy, ipsilateral INO, and ipsilateral peripheral facial nerve palsy can occur together in EHS. A vascular lesion is the most common cause, but a thorough workup is needed to exclude other etiologies.

It is important to recognize the clinical features of this condition in order to start prompt treatment. Rehabilitation and physical therapy play a crucial role in recovery. In cases involving infarcts, recovery is contingent upon the ability of the affected area to be salvage. Isolated cases of diplopia and facial palsy are associated with a better prognosis.
